# An annotated image dataset of pests on different coloured sticky traps acquired with different imaging devices

**DOI:** 10.1016/j.dib.2024.110741

**Published:** 2024-07-14

**Authors:** Song-Quan Ong, Toke Thomas Høye

**Affiliations:** a Department of Ecoscience Aarhus University, C. F. Møllers Allé 8, DK-8000 Aarhus C, Denmark; b Institute for Tropical Biology and Conservation, Universiti Malaysia Sabah, Jalan UMS, 88400 Kota Kinabalu, Sabah Malaysia; c Arctic Research Centre, Aarhus University, Ole Worms Allé 1, DK-8000 Aarhus C, Denmark

**Keywords:** Glue trap, Insect monitoring, Integrated pest management, Automated pest monitoring

## Abstract

The sticky trap is probably the most cost-effective tool for catching insect pests, but the identification and counting of insects on sticky traps is very labour-intensive. When investigating the automatic identification and counting of pests on sticky traps using computer vision and machine learning, two aspects can strongly influence the performance of the model – the colour of the sticky trap and the device used to capture the images of the pests on the sticky trap. As far as we know, there are no available image datasets to study these two aspects in computer vision and deep learning algorithms. Therefore, this paper presents a new dataset consisting of images of two pests commonly found in post-harvest crops – the red flour beetle (*Tribolium castaneum*) and the rice weevil (*Sitophilus oryzae*) – captured with three different devices (DSLR, webcam and smartphone) on blue, yellow, white and transparent sticky traps. The images were sorted by device, colour and species and divided into training, validation and test parts for the development of the deep learning model.

Specifications Table

**Table:** 

Subject	Biological Sciences/Computational Biology.
Specific subject area	The study of an automated pest monitoring system using computer vision and machine learning.
Type of data	Image.
Data collection	In a real-world situation, the end user would likely use different devices to take pictures of different coloured sticky traps. Therefore, we aimed to generate image data of pests based on yellow, blue, white, transparent and mixed four-colour sticky traps by using three different capturing devices - digital single-lens reflex (DSLR) cameras, webcams and mobile phones. The images were taken in a 30 × 30 × 30 cm photo studio box with white light illumination. The equipment was stabilised with a tripod, which was placed upside down and pointed at the box from above. The images were taken individually and saved in JPEG format. The image files were annotated and organized according to their background colour and the equipment used for the images.
Data source location	Institute for Tropical Biology and Conservation, Universiti Malaysia Sabah and Department of Ecoscience, Aarhus University, Denmark
Data accessibility	Repository name: Annotated images of pests on different coloured sticky traps with different imaging devices Data identification number: 10.6084/m9.figshare.23617383.v2 Direct URL to data: https://doi.org/10.6084/m9.figshare.23617383.v2
Related research article	–

## 1 Value of the Data

• As different insect species are attracted to different colored sticky traps, a dataset of pests that respond to different colored sticky traps would be valuable for the study of computer vision and machine learning models.
• Different imaging systems or devices such as smartphones and digital single-lens reflex (DSLRs) cameras are a common challenge for computer vision – what impact does the image quality have on the model's performance? The dataset consists of images taken with three different devices under laboratory and field conditions.
• The dataset is primarily used to evaluate a machine learning model for an automated monitoring system, in terms of the color of the sticky traps and the devices used to capture the images.
• Users such as field biologists, pest controllers or insect conservationists using an automated monitoring system could use the dataset to calibrate a machine learning model and investigate its suitability before using it in an automated pest monitoring system.

## 2 Background

Sticky traps are a valuable and non-toxic method in pest control and insect monitoring programmes [1]. These traps come in different colours to catch different types of pests. Computer vision and deep learning algorithms offer an excellent alternative for automatic detection and counting of pests on sticky traps [2]; however, two crucial elements need to be considered: Do imaging devices introduce significant variation in data acquisition for developing machine learning models, and which devices are best suited for imaging? Secondly, what is the influence of the colour of the sticky trap? These two questions also arise frequently when data scientists attempt to develop automated insect monitoring systems. In this paper, we present an image dataset in which two common store product pests – the red flour beetle (*Tribolium castaneum)* and the rice weevil (*Sitophilus oryzae*) - were captured on four different coloured sticky traps, namely yellow, blue, white and transparent, using three different devices – a digital single-lens reflex camera (DSLR), a phone and a webcam. The images were annotated with the respective devices, colours and species.

## 3 Data Description

**Fig. 1:**
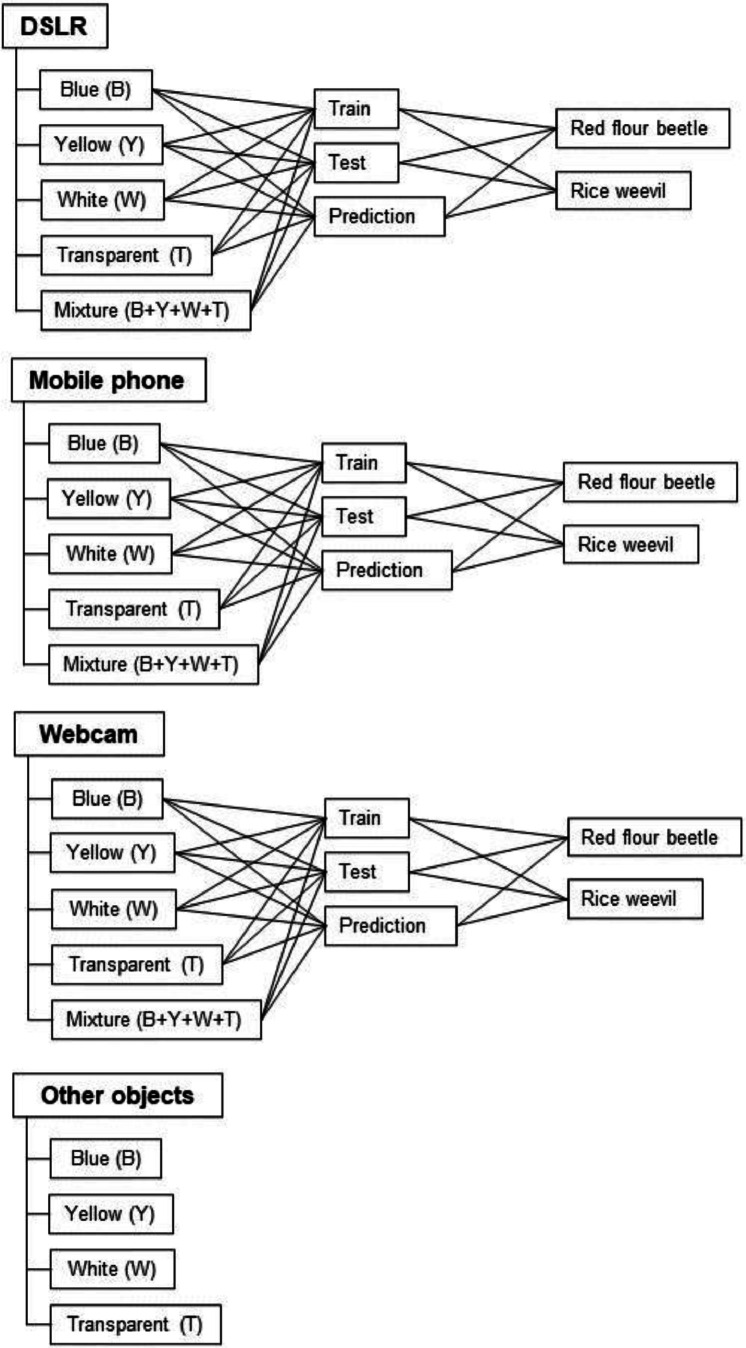
The general workflow to record the dataset and organised it into the labelled classes.

**Fig. 2:**
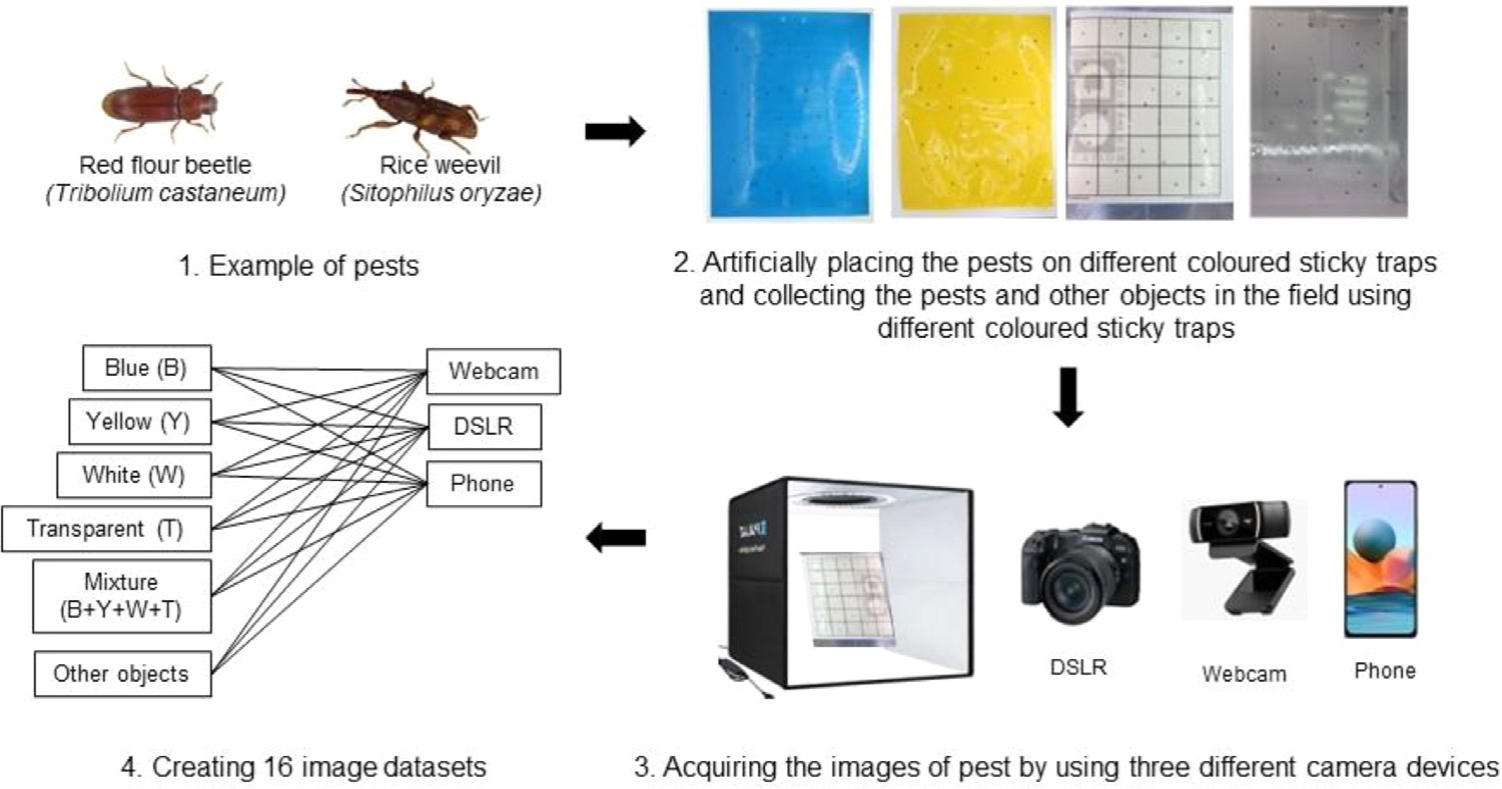
General workflow to record the dataset and organised into the annotated classes.

Fig. 1 illustrates the organization of the images according to their annotation and Table 1 shows the distribution of the number of images by class. After the images were captured with three imaging devices, they were formatted to 224×224 pixels for the usual training of the deep learning model. There are two main factors for the study - the type of imaging devices and the color of the sticky trap. The user can choose to train the model based on the label of the devices – three classes - or the label of the color of the sticky traps – five classes. The data was split for training, testing and prediction of the model: Training (70 %), Test (15 %) and Prediction (15 %). To avoid overfitting the model, augmentation was performed after splitting the data to avoid overlap of the training data in the test and prediction sets. Before developing the deep learning models, a series of data augmentation techniques were performed, rotating all images by 0, 90, 180 and 270° (Fig. 2).

**Table 1:** The number of images according to the classes.

Colour of sticky trap	Insect species	Imaging devices		
		DSLR	Phone	Webcam
Blue (B)	Red flour beetle	300	304	320
	Rice weevil	300	304	280
Yellow (Y)	Red flour beetle	300	304	240
	Rice weevil	300	304	240
White (W)	Red flour beetle	300	276	304
	Rice weevil	300	276	304
Transparent (T)	Red flour beetle	300	236	288
	Rice weevil	300	236	288
Mixture (BYWT)	Red flour beetle	300	292	300
	Rice weevil	300	292	292
Other objects*	Ants, Rice grain, Flour lump, Oat fragment	348		

⁎ Other objects datasets were used to evaluate the model with respect to the false positive rate (FPR) containing objects in the vicinity of the target pest. This dataset was also mainly used to evaluate the effects of background colour and device diversity.

## 4 Experimental Design, Materials and Methods

Fig. 1 illustrates the general workflow for collecting the dataset and classifying it into the annotated classes. To investigate the effects of background colour and imaging devices, the image dataset, which consisted of two common store product pests – the red flour beetle (*Tribolium castaneum*) and the rice weevil (*Sitophilus oryzae*) - was captured on four different coloured sticky traps, namely yellow, blue, white and transparent, using three different devices – a digital single-lens reflex camera (DSLR), a phone and a webcam. The images were formatted in a JPEG file and converted to 224 × 224 pixels to be used for the development of deep learning models or Convolutional Neural Network (CNN). The images have a resolution of 96 dpi. The images have been annotated with the respective devices, colours and species. They are organised in three main folders by device: Within the devices, the files are ordered by sticky trap colour, and within each folder, the data is divided into training, test and prediction sets.

### 4.1 Resources on insects, sticky traps and devices

We simulated the insect on the sticky trap using two common storage pests: the red flour beetle (*Tribolium castaneum*) and the rice weevil (*Sitophilus oryzae*), which were bred at the insect laboratory of the Institute of Tropical Biology and Conversation (ITBC), University Malaysia Sabah. Both insect colonies were maintained at a temperature of 25±2 °C and 70±5 % humidity. The red flour beetle was reared in rolled oat flakes and the rice weevil in jasmine rice grains. The 5- to 6-week-old adult beetles were used for the experiment. The beetles were separated from the breeding media and killed with 95 % ethanol. The insects were artificially introduced into the sticky trap using tweezers. Each colour consisted of 15 red flour beetles or rice weevils, and five replicates of each colour were prepared. All sticky traps were two-sided sticky traps measuring 25 cm × 20 cm (CityFarm®, Malaysia). In addition, we placed a total of eight sticky cards (two cards per colour) in the storage room of a food marker to collect possible target pests and other objects that could be classified as false positives and added the images of pests and other objects to the data sets. The taxonomy of the insects was identified and validated by two taxonomists. The adult stage of the insect was used for image capture and the labelling of the dataset was set to species level.

### 4.2 Data acquisition

To simulate the real situation where the end user could use different devices to capture images of different coloured sticky traps. Therefore, we aimed to generate pest image data based on yellow, blue, white, transparent and mixed four-colour sticky traps by using three different capturing devices - digital single-lens reflex (DSLR) cameras, webcams and a mobile phone with the technical details given in Table 2. Images were captured according to the instructions provided by Ong et al. [3]. Briefly described, the images were taken in a 30 × 30 × 30 cm photo studio box with white light illumination. The equipment was stabilised with a tripod placed upside down and pointed at the box from above. The images were taken individually and saved in JPEG format. The image files were labelled and sorted according to their background colour and the equipment used for the images. Table 3 shows the description and an example of the annotated images.

**Table 2:** Technical specification of the devices and setting that used to acquire the images.

Type of imaging device	DSLR	Phone	Webcam
Brand and model	Canon EOS RP	Xiaomi Redmi note 9 Pro	Logitech C922 Pro
Camera sensor spec	26.2MP Full-Frame CMOS Sensor	64 MP, f/1.9, 26 mm (wide), 1/1.72″, 0.8 µm, PDAF	2 MP, CMOS 1/2.7
Lens	Tamron SP AF 90 mm f/2.8 Di Macro	–	–
Technical setting	ISO 800 auto white balance	ISO 800 auto white balance	Full 1080p at 30fps Auto white balance

**Table 3:** Description and example of annotated classes of flies.

## Limitations

The dataset contains a pre-processed file of data augmented with four degrees of rotation – 0°, 90°, 180°, 270° - and divided into a training set, a test set, and a prediction set to evaluate model performance. Therefore, the file directory in Figshare [4] can be used directly as a URL and imported into the programming environment. Nevertheless, the data set has some limitations (see below):

1. Too many versions of the imaging device model. When creating the dataset, only one DSLR model (Canon EOS RP), one phone model (Xiaomi Redmi Note 9 Pro) and one webcam model (Logitech C922 Pro) were used, which differ significantly in terms of sensor size and operating system.
2. The dataset consists of only two beetle species. The dataset consists of two common post-harvest pests - the red flour beetle (*Tribolium castaneum*) and the rice weevil (*Sitophilus oryzae*); however, other visually similar beetles such as *Tribolium confusum* or *Sitophilus granarius* are not included in the dataset.
3. Reflections from the camera flash on some images. About 10 % of the images were taken with the flash switched on to simulate the real situation of users (using the final product of the deployed model, e.g. apps) when predicting the pest. This could potentially affect the performance of the detection model.

## Ethics Statement

We, the authors, hereby confirm that we have read and comply with the ethical requirements for publication in Data in Brief. Furthermore, we affirm that the present work does not involve human subjects, animal testing, or data collected on social media platforms. We have conducted our research in accordance with all applicable ethical guidelines and regulations*.*

## CRediT authorship contribution statement

**Song-Quan Ong:** Conceptualization, Methodology, Software, Validation, Formal analysis, Investigation, Resources, Data curation, Writing – original draft, Writing – review & editing, Visualization. **Toke Thomas Høye:** Conceptualization, Validation, Investigation, Writing – review & editing, Supervision, Project administration, Funding acquisition.

## Data Availability

Annotated images of pests on different coloured sticky traps with different imaging devices (Original data) (Figshare).
